# Endoscopic minimally invasive modified Bentall procedure with sutureless valve through right anterior minithoracotomy

**DOI:** 10.1016/j.xjtc.2026.102303

**Published:** 2026-03-06

**Authors:** Ali El-Sayed Ahmad, Saad Salamate, Marwan Hamiko, Ali Bayram, Kaveh Eghbalzadeh, Farhad Bakhtiary

**Affiliations:** Department of Cardiac Surgery, University Hospital Bonn, Bonn, Germany

**Figure:**
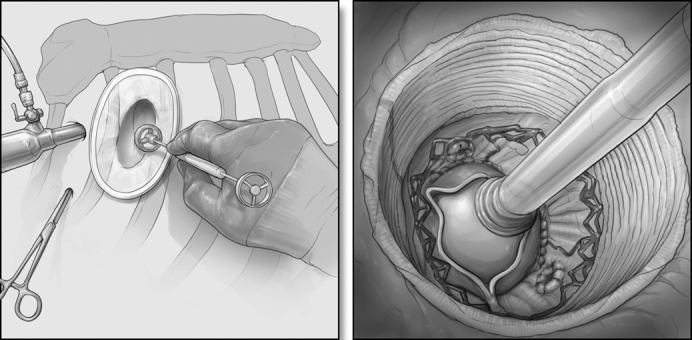
Endoscopic modified Bentall procedure with sutureless aortic valve prosthesis.

**Central Message**

Endoscopic right anterior minithoracotomy enables bioprosthetic Bentall using a Valsalva graft, and sutureless valve deployment within the graft simplifies valve implantation.

A Bentall procedure involves hand-sewn composite grafts and is recognized for its time-consuming and technical complexity.^1^ The modular valve-conduit construct uses a sutureless valve (SV) prosthesis to simplify the conventional Bentall procedure and reduce operative times.^2^ Endoscopic cardiac surgery represents a significant advancement in minimally invasive cardiac techniques, minimizing surgical trauma and facilitating enhanced postoperative recovery. This novel approach represents a significant step forward in the evolution of the endoscopic Bentall procedure, combining the advantages of minimal-access techniques with the efficiency of rapid-deployment valve systems.

## Operative Techniques

Clinical data and early postoperative outcomes of the 14 patients undergoing this novel procedure were collected after obtaining approval by the ethics committee of the University of Bonn (no. 464/22, approved on December 28, 2022) and written informed consent from all patients to publish this paper.

In this series, patients presented with an aneurysm of the ascending aorta and/or aortic root with concomitant aortic valve stenosis or regurgitation, documented by computed tomography and echocardiography, and were considered candidates for a totally endoscopic bioprosthetic Bentall procedure through a right anterior minithoracotomy. Patients with acute or chronic aortic dissection, redo cardiac surgery, or active endocarditis, patients scheduled for valve-sparing root replacement (David procedure), and patients requiring additional concomitant procedures (eg, coronary artery bypass grafting or other valve surgery) were excluded. These 14 cases represented 25% of our institutional aortic root replacement/Bentall volume from the last year.

**Figure E1:**
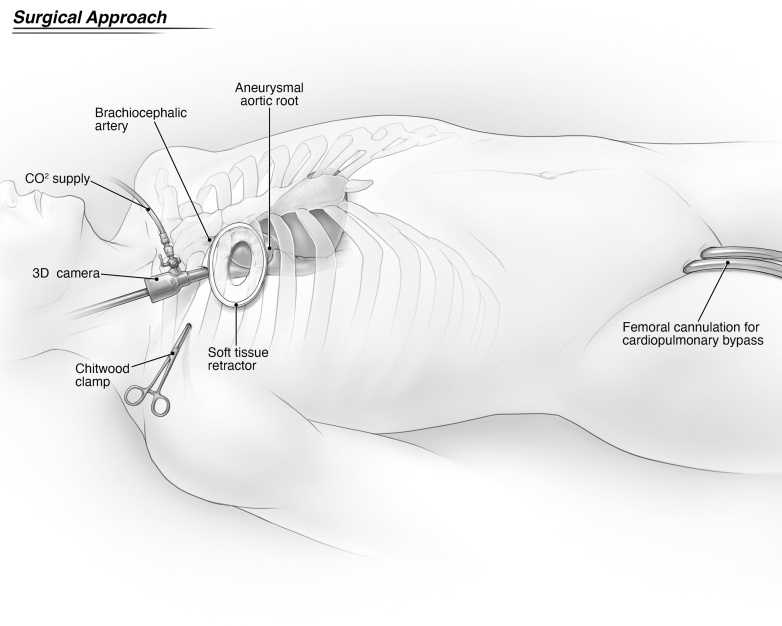
Overview of the surgical field of a right anterior minithoracotomy, with peripheral percutaneous cannulation for cardiopulmonary bypass.

A detailed step-by-step description of our approach to peripheral cannulation as well as the surgical access via a right anterior minithoracotomy have been previously published.^3^ The complete procedure is shown in Video 1, and surgical access and peripheral cannulation are shown in Figure E1.

**Figure 1:**
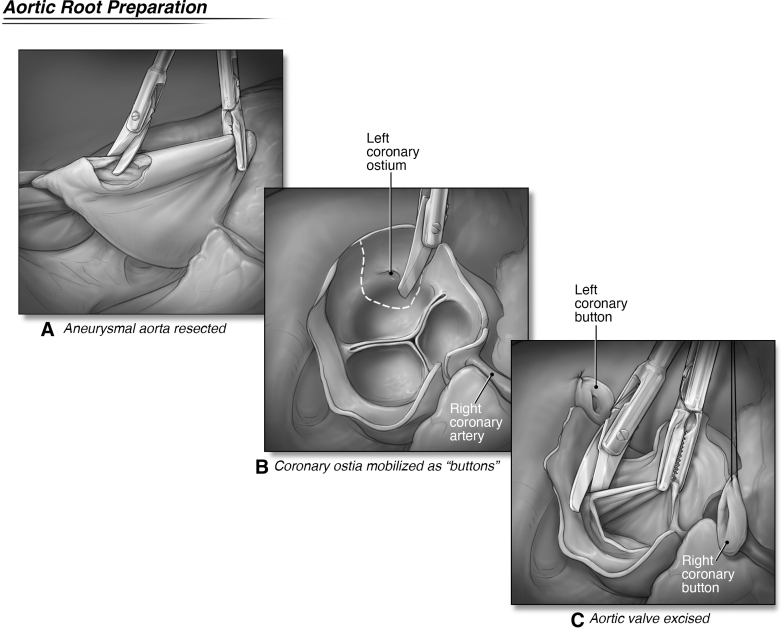
Aortic root preparation. Resection of the aneurysmal ascending aorta (A), mobilization of coronary “buttons” (B), followed by excision of the native aortic valve (C).

**Figure 2:**
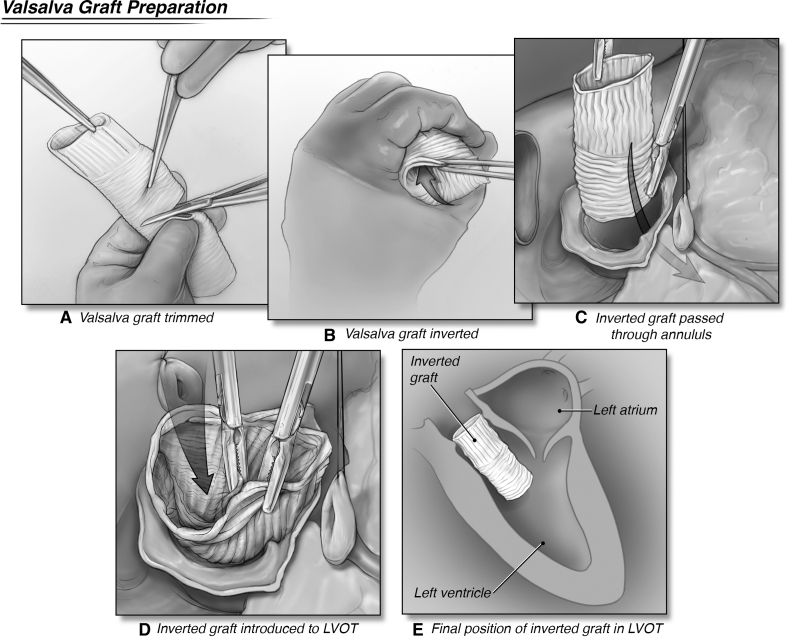
Valsalva graft preparation. The Valsalva graft is trimmed (A), inverted (B), and passed through the aortic annulus (C), where it is positioned in the left ventricular outflow tract (D and E).

In summary, after pericardiectomy and crossclamping of the aorta, the aneurysmal ascending aorta is resected, leaving a 2-cm wide margin to the aortic clamp distally and a 3-mm rim at the level of the aortic sinuses (Figure 1, *A*). The aortic valve is excised entirely and both coronary ostia are carefully mobilized as “buttons” and stabilized with stay sutures (Figure 1, *B* and *C*). A Vascutek Valsalva graft (Gelweave Valsalva; Vascutek Terumo Inc) is then trimmed, inverted, positioned in the left ventricular outflow tract (Figure 2).

**Figure 3:**
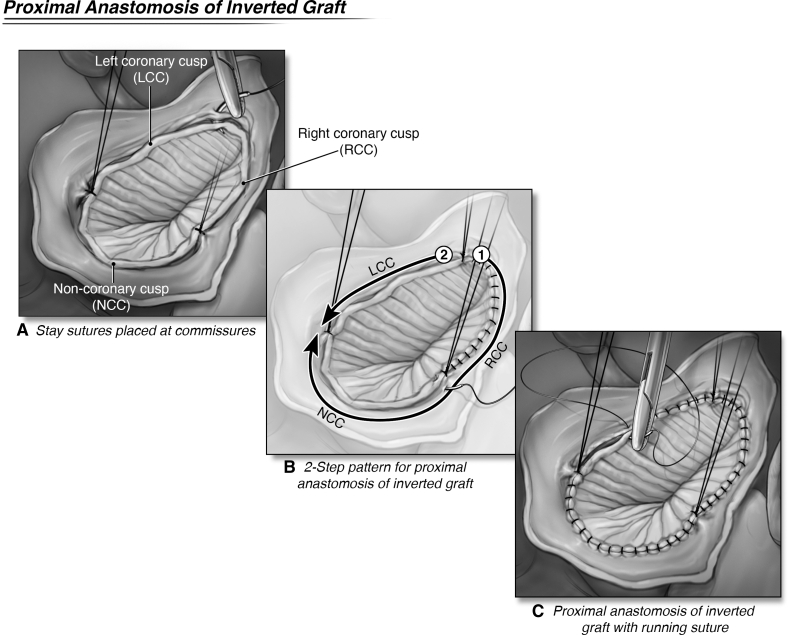
Proximal anastomosis of the inverted Vascutek Valsalva graft starting with 3 commissural stay sutures (A), followed by proximal anastomosis of the graft in 2 steps (B and C)

**Figure 4:**
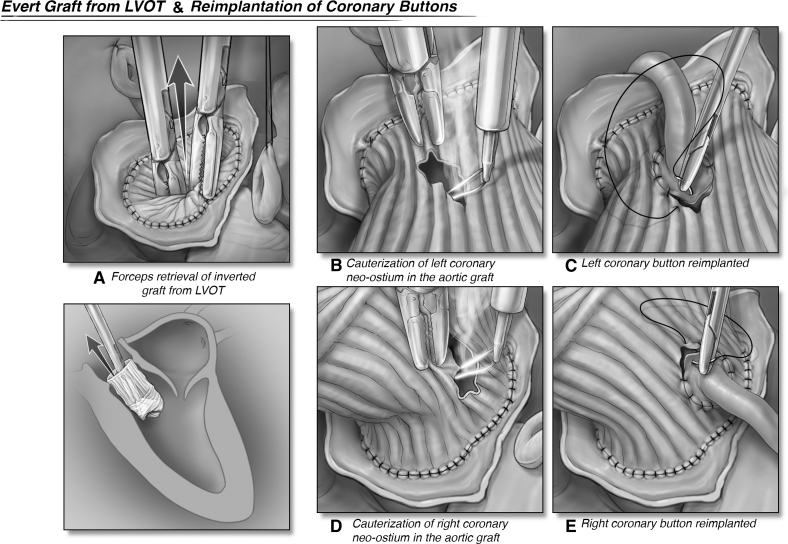
A, Eversion of the Valsalva graft from the left ventricular outflow tract. The inverted graft is retrieved using forceps and everted back to its normal configuration. B and D, cauterization of neo-ostia in the aortic graft. C and E, Reimplantation of the coronary buttons.

The Valsalva graft is fixed to the aortic annulus with the help of 3 commissural stay sutures (Figure 3, *A*) and is then secured to the aortic annulus using a 4-0 polypropylene running suture beginning from the right-left commissure to the left noncoronary commissure in 2 steps, first covering the right and noncoronary cusp in a clockwise fashion, then the left coronary cusp in an anticlockwise fashion (Figure 3, *B* and *C*). The graft is then externalized from the left ventricular outflow tract through eversion (Figure 4, *A*).

**Figure 5:**
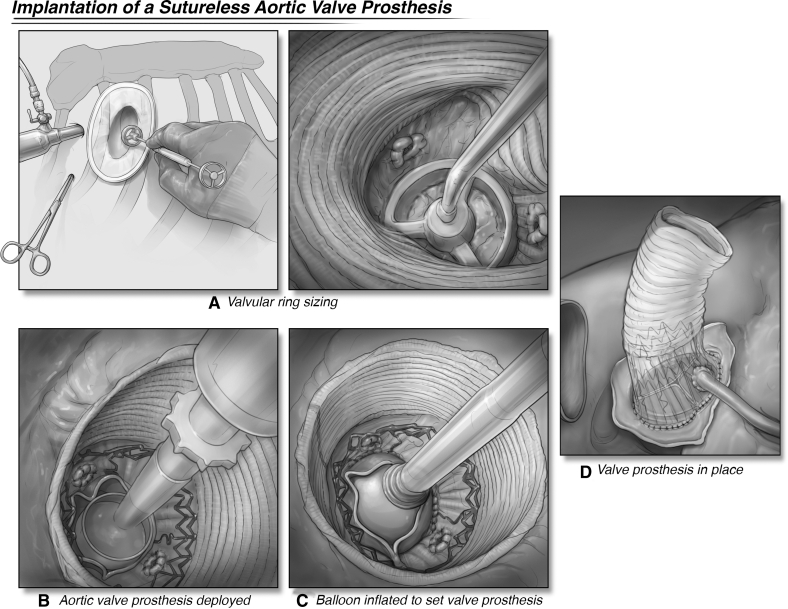
Sizing (A) and implantation of a sutureless aortic valve prosthesis through a right anterior minithoracotomy (B and C). D, The Perceval valve is shown correctly fit and aligned.

Openings for the left and right coronary arteries are precisely created in the graft using electrocautery (Figure 4, *B* and *D*). The coronary buttons are reimplanted into the graft with 4-0 polypropylene running sutures (Figure 4, *C* and *E*). After measuring the aortic valve annulus, a Perceval sutureless valve (Corcym UK Limited) is implanted within the Valsalva graft (Figure 5).

**Figure E2:**
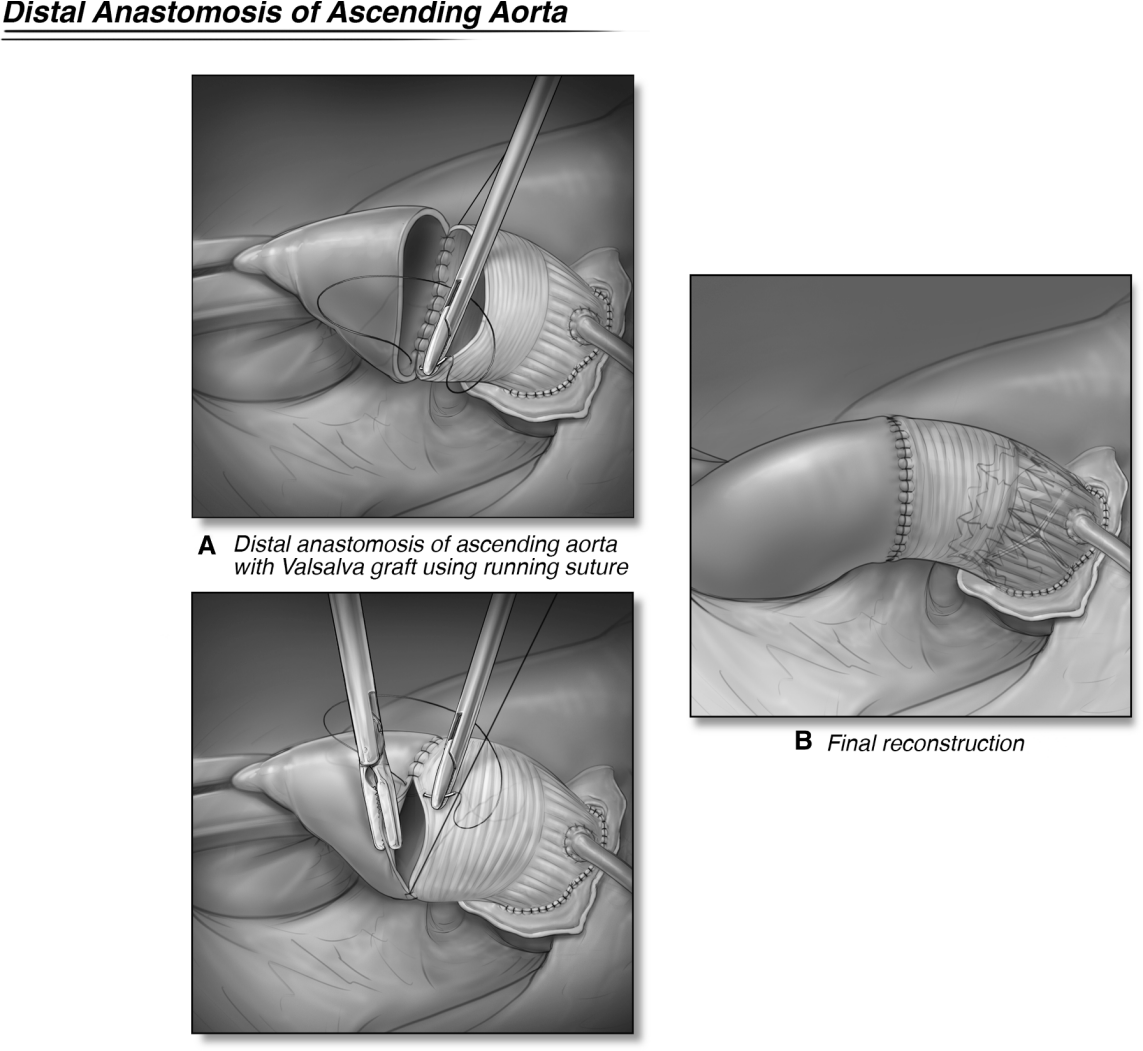
Distal anastomosis of the ascending aorta.

After the length of the vascular graft is determined, its distal end is anastomosed to the native ascending aorta using a 3-0 polypropylene running suture (Figure E2). Finally, cardiopulmonary (CPB) weaning, decannulation, and arterial closure using the MANTA vascular closure system (Teleflex) are performed.

## Comment

Our minimally invasive modification of the Bentall procedure combines the advantages of endoscopic surgery and the SV technology. On the one hand, the endoscopic approach avoids complications associated with sternotomy, such as wound infections, sternal instability, and prolonged healing time.^2^^,^^4^ On the other hand, the use of SV reduces the procedural complexity, aortic crossclamping time, and CPB duration while maintaining excellent hemodynamic results and durability.^5^ The adaptation of SV in endoscopic cardiac surgery mitigates the technical challenges and reduces morbidity and mortality.

Our novel approach was applied to 14 patients with aneurysm of the ascending aorta and aortic root with aortic valve pathology (Table E1). Mean CPB and aortic crossclamping times were 101.2 ± 31.3 minutes und 71.2 ± 21.8 minutes, respectively. There was no incidence of rethoracotomy, reintervention, or stroke, and 30-day mortality was 0. Other outcomes are detailed in Table E2. With the demonstration of lower CPB and crossclamping durations with similar outcomes to our previously published results of endoscopic Bentall procedure with a sutured valvular prosthesis,^3^ these preliminary results indicate that the use of an SV in endoscopic Bentall procedure is a safe and feasible approach in select patients.

## Conclusions

The endoscopic Bentall procedure with an SV represents a promising innovation for managing complex aortic root pathologies. By potentially reducing the operative time and complexity of the procedure, this approach offers an effective and safe alternative to traditional methods, particularly in high-risk patients.

## Conflict of Interest Statement

Dr Bakhtiary reports a relationship with Edwards Lifesciences, Medtronic, Corcym, and Abbott that includes consulting or advisory and speaking and lecture fees. Dr Bakhtiary reports a relationship with LSI that includes speaking and lecture fees. All other authors reported no conflicts of interest.

The *Journal* policy requires editors and reviewers to disclose conflicts of interest and to decline handling or reviewing manuscripts for which they may have a conflict of interest. The editors and reviewers of this article have no conflicts of interest.

## Figures and Tables

**Table E1:** Patient characteristics

Baseline data	N = 14
Age, y	66.5 ± 9.3
Male	7 (50%)
BMI	28.3 ± 4.6
Aortic regurgitation >I	5 (35.7%)
Aortic stenosis	6 (42.9%)
Bicuspid aortic valve	6 (42.9%)
Ejection fraction, %	53.7 ± 7.3
Hypertension	10 (71.4%)
Diabetes mellitus	3 (21.4%)
Coronary artery disease	4 (28.6%)
Chronic obstructive pulmonary disease	4 (28.6%)
Peripheral arterial vascular disease	3 (21.4%)
Previous stroke	3 (21.4%)
Atrial fibrillation	5 (35.7%)

Values are expressed as mean ± standard deviation or as number and percentage. *BMI*, Body mass index.

**Table E2:** Outcomes

Outcome	N = 14
CPB time, min	101.2 ± 31.3
Aortic crossclamp time, min	71.2 ± 21.8
Bleeding	0 (0%)
Pericardial effusion	0 (0%)
Rethoracotomy	0 (0%)
Myocardial infarction	0 (0%)
Conversion to sternotomy	0 (0%)
Renal failure	0 (0%)
Stroke	0 (0%)
Delirium	1 (7.1%)
Atrial fibrillation	2 (14.3%)
Pacemaker implantation	0 (0%)
Wound infection	0 (0%)
In-hospital mortality	0 (0%)
30-d mortality	0 (0%)
Reintervention	0 (0%)
Postoperative transfusion RBC concentrate	1 ± 1.3
Postoperative transfusion FFPs	0
Postoperative transfusion platelets	0
Ventilation time in h	5.3 ± 7.4
ICU time in h	34.2 ± 21.1
Length of hospital stay in d	7.2 ± 1.8

Values are expressed as mean ± standard deviation or as number and percentage. *CPB*, Cardiopulmonary bypass; *RBC*, red blood cell; *FFP*, fresh frozen plasma; *ICU*, intensive care unit.

## Supplementary Data

Video 1 Endoscopic minimally invasive modified Bentall procedure with sutureless valve through right anterior mini-thoracotomy. Video available at: https://www.jtcvs.org/article/S2666-2507(26)00110-0/fulltext.
