# Supplementary figures and images for: Endoscopic minimally invasive modified Bentall procedure with sutureless valve through right anterior minithoracotomy
Source: JTCVS Tech. 2026 Mar 6;37:102303. doi: 10.1016/j.xjtc.2026.102303 (PMC13261195; doi:10.1016/j.xjtc.2026.102303)

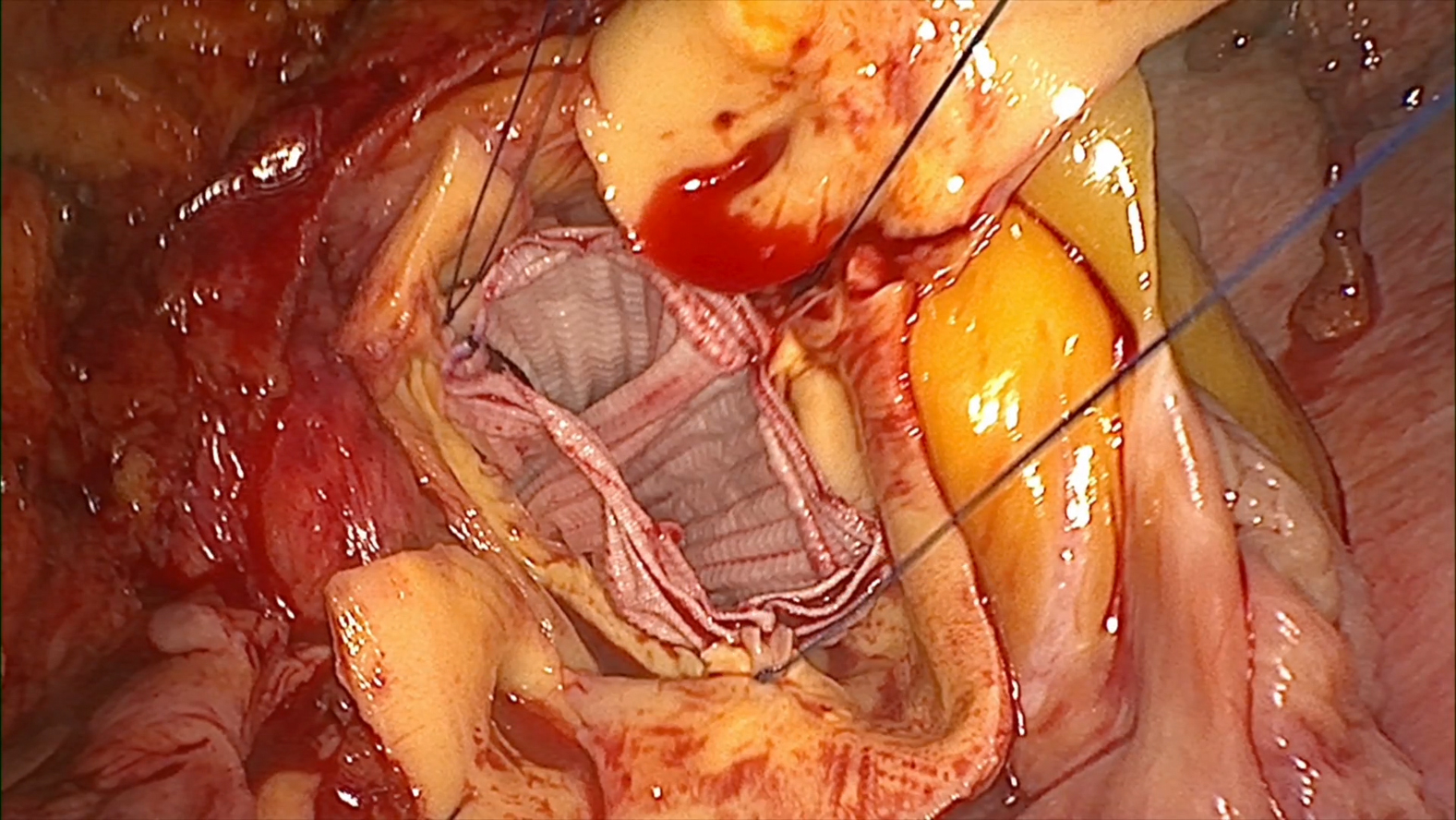

Supplement: Video 1 — Endoscopic minimally invasive modified Bentall procedure with sutureless valve through right anterior mini-thoracotomy. Video available at: https://www.jtcvs.org/article/S2666-2507(26)00110-0/fulltext. [file fx2.jpg]
